# The NIHR collaboration for leadership in applied health research and care (CLAHRC) for greater manchester: combining empirical, theoretical and experiential evidence to design and evaluate a large-scale implementation strategy

**DOI:** 10.1186/1748-5908-6-96

**Published:** 2011-08-23

**Authors:** Gill Harvey, Louise Fitzgerald, Sandra Fielden, Anne McBride, Heather Waterman, David Bamford, Roman Kislov, Ruth Boaden

**Affiliations:** 1 Manchester Business School, University of Manchester, Booth Street West, Manchester, M15 6PB, UK; 2 School of Nursing, Midwifery and Social Work, University of Manchester

## Abstract

**Background:**

In response to policy recommendations, nine National Institute for Health Research (NIHR) Collaborations for Leadership in Applied Health Research and Care (CLAHRCs) were established in England in 2008, aiming to create closer working between the health service and higher education and narrow the gap between research and its implementation in practice. The Greater Manchester (GM) CLAHRC is a partnership between the University of Manchester and twenty National Health Service (NHS) trusts, with a five-year mission to improve healthcare and reduce health inequalities for people with cardiovascular conditions. This paper outlines the GM CLAHRC approach to designing and evaluating a large-scale, evidence- and theory-informed, context-sensitive implementation programme.

**Discussion:**

The paper makes a case for embedding evaluation within the design of the implementation strategy. Empirical, theoretical, and experiential evidence relating to implementation science and methods has been synthesised to formulate eight core principles of the GM CLAHRC implementation strategy, recognising the multi-faceted nature of evidence, the complexity of the implementation process, and the corresponding need to apply approaches that are situationally relevant, responsive, flexible, and collaborative. In turn, these core principles inform the selection of four interrelated building blocks upon which the GM CLAHRC approach to implementation is founded. These determine the organizational processes, structures, and roles utilised by specific GM CLAHRC implementation projects, as well as the approach to researching implementation, and comprise: the Promoting Action on Research Implementation in Health Services (PARIHS) framework; a modified version of the Model for Improvement; multiprofessional teams with designated roles to lead, facilitate, and support the implementation process; and embedded evaluation and learning.

**Summary:**

Designing and evaluating a large-scale implementation strategy that can cope with and respond to the local complexities of implementing research evidence into practice is itself complex and challenging. We present an argument for adopting an integrative, co-production approach to planning and evaluating the implementation of research into practice, drawing on an eclectic range of evidence sources.

## Background

Evidence-based healthcare has featured as a policy concern in many healthcare systems over the last decade, driven by a growing recognition that healthcare delivery does not always reflect what is known to be best practice. Studies suggest that up to thirty to forty per cent of patients do not receive care complying with current scientific evidence [1,2]. Responding to these concerns, attempts have been made to find ways to narrow the research-practice gap and ensure that research is translated into clinical practice and service delivery as effectively and efficiently as possible.

In the UK, the Cooksey Report on research funding [3] identified two gaps in the translation of health research, namely translating ideas from basic and clinical research into the development of new products and approaches to treatment of disease and illness, and implementing those new products and approaches into clinical practice. Subsequently, the High Level Group on Clinical Effectiveness [4] highlighted: the need for a range of measures to narrow the gap between evidence and implementation, including measures to promote local ownership of the clinical effectiveness agenda, with clinicians and managers working in partnership; the need for the health service to make better use of the skills and expertise available in higher education organizations; the need for increased understanding of the mechanisms that encourage the adoption of new interventions; and the need for more research on organizational receptivity.

Responding to these policy recommendations, Collaborations for Leadership in Applied Health Research and Care (CLAHRCs) have been established in England in an attempt to create closer working between the health service and higher education, and thus narrow the gap between research and its implementation in practice. In total, nine Collaborations were funded by the National Institute for Health Research (NIHR) for a five year period, starting in October 2008, each with three related objectives, namely: conducting high-quality applied health research; implementing the findings from research in clinical practice; and increasing the capacity of National Health Service (NHS) organizations to engage with and apply research. Protocols from some of the CLAHRCs have previously been published in *Implementation Science *[5,6].

Within Greater Manchester (GM), the CLAHRC is a partnership between the University of Manchester and twenty NHS trusts. It has a five-year mission to improve healthcare and reduce health inequalities for people with cardiovascular conditions (diabetes, chronic kidney disease, stroke, and heart failure). There are two key strands of activity within the CLAHRC: one undertaking applied health research to support patient self-management and improve the quality of care for people with chronic vascular disease; the second, an implementation programme that is focused on implementing research evidence relevant to clinical areas where a known gap exists between current practice and established best practice, as indicated, *e.g*., by research, systematic reviews, and clinical guidelines.

The GM CLAHRC implementation programme is comprised of four implementation themes focusing, respectively, on the four cardiovascular conditions of chronic kidney disease, heart failure, diabetes, and stroke. Each of the themes has designed an individual implementation project, dependent on the specific clinical issue being addressed, stakeholders engaged, and other contextual factors. However, it was recognised from the outset that all GM CLAHRC implementation activities should be evidence- and theory-informed and context-dependent, and that they should be underpinned by the same general founding principles. This was seen to be important for the following reasons: first, to maximise the likelihood of successful implementation; second, to generate learning about the implementation process and add to the knowledge base about how best to get evidence into healthcare practice; and, finally, to enhance the interconnectedness of the implementation themes and integrity of the programme as a whole.

This paper outlines the GM CLAHRC approach to designing and evaluating a large-scale, evidence-informed, theory-driven, and context-sensitive implementation programme. It describes the types of evidence about implementation that have been utilised; formulates eight core principles underpinning the GM CLAHRC implementation strategy; and describes how these principles have influenced the selection of theoretical and operational models, the development of organizational structures and processes, and the approach to evaluation within the implementation programme. Because the main purpose of the paper is to describe general theoretical foundations upon which the GM CLAHRC implementation programme has been built, it will not specifically discuss how these principles have been interpreted and adapted by individual implementation projects within the programme; nor will it analyse strengths and weaknesses of the approach and reflect on its overall effectiveness. These aims are being addressed by ongoing internal and external evaluations of the GM CLAHRC and will be reported in subsequent papers.

## Design

### Combining evidence from different sources to inform implementation

In designing the implementation programme, we have explicitly taken an approach that aims to integrate theoretical, empirical, and experiential evidence about implementation interventions in healthcare (Table 1). Before describing each of the three types of evidence in more detail and presenting the rationale for selecting this integrated approach, it is worth making several clarifying comments. In contrast to a biomedical tradition which predominantly uses the term 'evidence' to refer to the findings of empirical research conducted in controlled conditions, it will be employed in this section in a broader sense, referring also to other forms of knowledge seen as credible from a social science perspective. In addition, it should be emphasized that we will be speaking here about knowledge relating to the effectiveness of certain implementation strategies and interventions, *i.e*., evidence about implementation, rather than the clinical knowledge about the effectiveness of certain diagnostic and treatment methods (in other words, the evidence to be implemented).

**Table 1:** Types of evidence to inform implementation [11]

Type of evidence	Description	How it contributes to knowledge
Theoretical	Ideas, concepts, and models used to describe the intervention, to explain how and why it works, and to connect it to a wider knowledge base and framework	Helps to understand the programme theories that lie behind the intervention, and to use theories of human or organizational behavior to outline and explore its intended working in ways that can be used to construct and test meaningful hypotheses and transfer learning about the intervention to other settings

Empirical	Information about the actual use of the intervention, and about its effectiveness and outcomes in use	Helps to understand how the intervention plays out in practice, and to establish and measure its real effects and the causality of relationships between the intervention and desired outcomes

Experiential	Information about people's experiences of the service or intervention, and the interaction between them	Helps to understand how people (users, practitioners, and other stakeholders) experience, view, and respond to the intervention, and how this contributes to our understanding of the intervention and shapes its use

In recent years, we have witnessed a growth of activity within the field of implementation research generating empirical knowledge about how best to implement evidence into practice. Empirical evidence produced by this research provides some useful starting points in the design of an implementation programme, indicating, *e.g*., interventions that have varying levels of effectiveness [7] (Table 2) and the ways in which research evidence is negotiated, contested, and possibly ignored during the processes of translation and implementation [8]. However, on its own, such empirical evidence is insufficient to plan a detailed implementation approach. Take, for example, systematic review evidence that suggests multi-faceted interventions are one of the most consistently effective ways to get evidence into practice [9]. While this provides an important starting point, there is little guidance on which combination of interventions to put together, how, and when. This is why it is useful to combine the empirical evidence with both theoretical evidence and experiential evidence from the field to produce knowledge that is 'fit for purpose' [10], *i.e*., tailored to the particular circumstances or situation in which implementation is to take place.

**Table 2:** Evidence of effectiveness for interventions to promote behavioural change among health professionals [7]

Consistently effective	Variable effectiveness	Little or no effect
• Educational outreach visits (for prescribing in North America) • Reminders (manual or computerised) • Multifaceted interventions (a combination that includes two or more of the following: audit and feedback, reminders, local consensus processes, or marketing) • Interactive educational meetings (participation of healthcare providers in workshops that include discussion of practice)	• Audit and feedback (or any summary of clinical performance) • The use of local opinion leaders (practitioners identified by their colleagues as influential) • Local consensus processes (inclusion of participating practitioners in discussions to ensure that they agree that the chosen clinical problem is important and the approach to managing the problem is appropriate) • Patient-mediated interventions (any intervention aimed at changing the performance of healthcare providers for which specific information was sought from or given to patients)	• Educational materials (distribution of recommendations for clinical care, including clinical practice guidelines, audiovisual materials, and electronic publications) • Didactic educational meetings (such as lectures)

Theoretical evidence is a growing area of interest within the field of implementation science, both in relation to changing the behaviour of individuals and at the organizational level to aid understanding of the broader set of economic, administrative, managerial, or policy-related factors that may influence implementation [11-13]. Theories are seen to provide a useful way of contextualising, planning, and evaluating implementation strategies that typically comprise multiple interventions targeted at different groups and different levels within an organization. Such informing theories may be drawn from a broad range of disciplines, including, *e.g*., psychology, organizational behaviour, social marketing, and organizational learning [12]. This is reflected in the eclectic, multi-theoretical approach to implementation which has been taken by the GM CLAHRC implementation programme and which will be discussed in more detail in subsequent sections of this paper.

We also draw on the experiential evidence of those involved in applying evidence in practice. This evidence, in turn, has several sources. First of all, some of the clinical, managerial, and academic members of the implementation team have undertaken considerable work within the field of implementation research and practice, and this pre-existing, practice-based knowledge has a significant role in shaping and refining the implementation programme as it develops and evolves. In addition, the GM CLAHRC implementation activities are also shaped by the experiential evidence collected from various external stakeholders from within the NHS, who possess valuable knowledge about contextual factors that need to be addressed in the process of implementing change. Last but not least, important experiential evidence is being acquired, negotiated, and translated into practice by the members of the CLAHRC implementation teams as part of 'learning by doing' in the course of implementing their projects [14].

Given the inherent complexity and context-dependent nature of the implementation process, as well as the insufficiency of empirical evidence about implementation, it becomes impractical to prioritise one type of knowledge over the others. As such, our approach to implementation is integrative, developmental, and reflective, making use of evidence about implementation that already exists and applying and refining this evidence as the work of the CLAHRC unfolds and progresses. Empirical, theoretical, and experiential evidence is collected and collated through reviewing relevant literature and sharing the collective knowledge and experience of implementation team members. This synthesis has led to the formulation of eight core principles that underpin the GM CLAHRC approach to implementation (Table 3) and are discussed in the following section.

**Table 3:** Core principles underpinning the Greater Manchester CLAHRC implementation approach

■ Evidence is broader than research
■ Good research is not enough to guarantee its uptake in practice
■ Rational/linear models are inadequate in planning and undertaking implementation
■ Acknowledgement of and responsiveness to the context of implementation
■ Tailored, multi-faceted approaches to implementation are needed
■ Importance of forming networks and building good relationships
■ Individuals are required in designated roles to lead and facilitate the implementation process
■ Integrated approach to the production and use of evidence about implementation

### Core principles underpinning implementation

#### Evidence is broader than research

Evidence derived from research is a central focus in initiatives such as CLAHRCs that are attempting to bridge the gap between research and decision making at the level of health service delivery and practice. However, it is equally important to be cognisant of empirical studies that demonstrate the complex, multi-faceted, and contested nature of evidence in the healthcare setting [15,16]. While rigorous techniques have been developed to increase the objectivity of research evidence (*e.g*., systematic review, technology appraisal, and clinical guideline development), studies suggest that in practice, healthcare professionals draw on and integrate a variety of different sources of evidence, encompassing both propositional and non-propositional knowledge. Sources of evidence that sit alongside research typically include knowledge derived from clinical experience, from credible colleagues, patients, clients, and carers, and from the local context or environment [17,18].

This experiential evidence presents a challenge to the traditional hierarchy of evidence within biomedical research, whereby 'gold standard' evidence is that derived from multiple, high-quality randomized controlled trials or systematic reviews. In practice, evidence relating to the effectiveness of interventions is considered alongside a range of other criteria, including, *e.g*., acceptability, accessibility, appropriateness, and fit with local priorities [11]. Thus, designing an implementation strategy that relies on a narrow definition of evidence as research (and ranking the strength of that research according to the research design) is likely to result in an approach that fails to acknowledge the complexity of decision making at the level of clinical practice and service delivery.

#### Good research is not enough to guarantee its uptake in practice

The multi-faceted nature of evidence has implications for the way in which research is implemented (or not) in practice. While some strategies for getting research into practice, such as evidence-based clinical guidelines, assume a direct or instrumental process of research utilisation [19], the reality in practice has been shown to be significantly more complex [15,20]. Dopson and Fitzgerald [8] draw on comparative data from a total of 48 primary case studies of the careers of evidence-based innovations and highlight the importance of sense-making and the enactment of evidence in practice, in order to translate research evidence from information to new knowledge that can influence practice change. Other researchers have similarly highlighted that research evidence, although crucial to improving patient care, may not on its own inform practitioners' decision-making [17,21], because of the need to translate and particularise evidence in order to make sense of it in the context of caring for individual patients [18]. Furthermore, at this more local level, other factors come into play, including the credibility of the evidence source and competing priorities, which may distort the types of evidence that individuals pay attention to [22].

#### Rational/linear models are inadequate in planning and undertaking implementation

Early models of evidence-based practice suggested a fairly straightforward, linear process of translating research into practice [23], where once evidence was reviewed and collated (*e.g*., in the form of systematic reviews or clinical guidelines), then processes of dissemination, continuing professional development, and clinical audit could be used to promote uptake of the research in practice. Linear models of research use typically view research and practice as two separate entities and emphasize the flow of knowledge from researchers to the practice community [19]. Another way of viewing these more linear models is as 'producer-push' approaches [24], with practitioners as the recipients of research.

However, experiences in practice and studies to evaluate the implementation of research into practice have repeatedly highlighted the complexity of the process, linked to factors such as the multi-faceted nature of evidence, the influence of contextual factors, and the mediating role of professional groups [8,25,26]. Subsequently, alternative models to implementing research evidence in practice have been developed that attempt to move beyond the linear approach to implementation. These include models which represent implementation as a more cyclical process, still comprising a sequence of key steps, but taking place within a process of repeating cycles (*e.g*., the knowledge-to-action cycle [27]), and dynamic models which attempt to represent the simultaneous interaction of a number of key factors in the implementation process (*e.g*., the Promoting Action on Research Implementation in Health Services (PARIHS) framework [20,28]). A recent review of models that have been developed to guide the knowledge transfer process [29] concludes that it is the interactive, multi-directional models of implementation that most accurately represent the knowledge transfer process in action.

#### Acknowledgements of and responsiveness to the context of implementation

Context can be defined as 'the environment or setting in which the proposed change is to be implemented' [20] and is shaped by a range of different factors at the macro, meso, and micro levels of health service delivery. Ferlie *et al*. [30] identify context as a crucial determinant of the career of an evidence-based innovation, highlighting the influence of factors at the micro level in determining the receptiveness of an organization to change, in particular, the engagement of clinical opinion leaders, the quality of relationships, change and project management capacity, senior management support, organizational complexity, and a climate of organizational learning. McCormack *et al*. [31] similarly note a range of contextual influences at the micro and meso organizational level that influence the uptake of research, including: the existence of clearly defined boundaries; clarity about decision making processes; clarity about patterns of power and authority; resources, information, and feedback systems; active management of competing 'force fields' that are never static; and systems in place that enable dynamic processes of change and continuous development [30].

Such features of the local context are clearly influenced by the prevailing organizational culture, by organizational history and politics, and by relationships with other key stakeholders in the health economy. In order to understand and manage the multiple contextual influences, implementation approaches need to have a clear strategy for assessing the organizational context in which implementation is to take place, including an assessment of the key stakeholders and their roles and relationships, and have sufficient flexibility to tailor implementation to fit the specific needs of the context.

#### The need for tailored, multi-faceted approaches to implementation

As previously noted, empirical evidence supports the use of multi-faceted interventions to implement research into practice [7]. However, the specific combination of different interventions within an implementation strategy (*e.g*., audit and feedback, reminders, educational events, opinion leaders, *etc*.) can be less clearly defined, because of the need to tailor interventions to the specific needs of the local setting or context. This poses a central challenge to the design of a strategy for implementation, namely, how to know which package of interventions to put together, for which setting, and at what point in time.

As highlighted in the point above, having a sound understanding and assessment of the context is crucial to the plan for implementation at a local level. However, having undertaken a detailed assessment of the context, the question is then one of how to address the various political, leadership, relationship, and cultural issues that may be identified as influencing the process of implementing evidence into practice. This is where multi-faceted approaches need to embrace both specific interventions to make evidence more accessible and amenable to key stakeholders and active roles that seek to negotiate the potential barriers and obstacles to implementation.

#### Importance of forming networks and building good relationships

Denis and Lehoux [32] identify three building blocks relating to the organizational use of knowledge, which they describe as knowledge as codification (focusing on the synthesis of knowledge in the form of clinical practice guidelines or quality indicators), knowledge as capabilities (in the form of organizational structures and processes to enable knowledge transfer) and knowledge as process (mechanisms to build relationships, create a greater sense of coherence and enhance problem-solving). It is in relation to this third building block, knowledge as process, that relationships and networks are particularly important. In turn, this links to some of the principles already outlined, such as the contingent nature of evidence and the influence of local context, and highlights the need for implementation strategies to take account of and engage the appropriate range of stakeholders. This includes stakeholders with a perspective on evidence (*e.g*., researchers, clinicians, commissioners, patients, and the public) and those with an influence on the context (*e.g*., managers, policy makers, clinicians, patient representatives).

Increasingly, as evidence is recognized to be situational and subject to a process of sense making before it is implemented, attention has turned to focus on learning theories that can help to understand and explain the processes by which knowledge is shared and learned. For example, theories such as communities of practice [33,34] have been applied to explore the different meanings that different professional groups ascribe to the same evidence and the complex process of integrating and constructing knowledge across disciplinary boundaries [8]. This poses a challenge to all CLAHRCs with the multiple groupings that are engaged in the initiative: academics and practitioners, managers and clinicians, commissioners and providers, professionals and the public, to name just a few. At both the planning stage of implementation and throughout the process of implementation and evaluation, this highlights the need for a collaborative approach, with time and effort being invested in building relationships and creating networks for learning and sharing information.

#### Individuals in designated roles to lead and facilitate the implementation process

The principles outlined so far highlight the need for flexible, collaborative, and multi-faceted approaches to implementation. Linked to the second building block identified by Denis and Lehoux [32]--knowledge as capabilities--structures and processes are required to enact the tailored, multi-dimensional approaches to implementation, taking account of and responding to contextual influences and the varied (and sometimes competing) needs and priorities of local stakeholders. In communities of practice theory, knowledge is described as 'sticky' at the boundary between communities, *e.g*., different professional groups. Three distinct strategies are proposed for overcoming such boundary issues: using people to act as knowledge brokers between different communities; making use of boundary objects or artefacts; and establishing boundary practices to promote interaction among the different communities [35,36].

Within the field of evidence-based healthcare, various roles have been identified to support and lead boundary spanning activities, including, *e.g*., educational outreach workers, academic detailers, knowledge brokers, opinion leaders, and facilitators [37-42]. While the roles vary in terms of the position of individuals in relation to the organization (internal or external), their role and source of influence (*e.g*., professional versus non-professional) and the range of methods and techniques they might use (social marketing, influencing, leadership, facilitation), they share a common feature of one or more individuals assuming an explicit role to enable the translation and uptake of research knowledge into practice. Given the size and complexity of the CLAHRC, the large number of organizations involved and the multiple communities that are represented, a number of different boundary spanning roles, structures, and processes are required.

#### Integrated approach to the production and use of evidence about implementation

A final principle underpinning the GM CLAHRC approach to implementation relates to the previously described complex, contested, and contingent nature of evidence and a growing awareness that co-production of knowledge enhances its relevance and transferability to practice [43,44]. The consequence of this is that we did not start out with a pre-planned, detailed, top-down programme of implementation activities that we were aiming to apply within CLAHRC. Rather, the specific implementation projects are guided by the ongoing co-production of knowledge by the GM CLAHRC team members, who are engaged in the sharing, negotiation and practical application of empirical, theoretical, and experiential evidence relevant to their work. This co-produced, integrated, and applied knowledge promotes practice-based learning about what works and what does not work in a given context. In turn, this model of co-production underpins the approach to evaluation within the GM CLAHRC, as outlined in subsequent sections of the paper.

#### Designing an implementation approach based on the core principles

From the starting point of the core principles outlined above, there are a number of key messages that emerge and which fundamentally influence the approach to implementation that we are adopting. First, it is clear that we cannot treat research evidence as a 'product' to be implemented; second, we have to understand and work with a wide range of individuals and organizations, each with their own local conditions, politics, and priorities; third, we require an approach that provides an overall structure, based on the underpinning principles, but allows for local flexibility; fourth, we have to have the right people in the right roles to maximize the chances of success; and finally, we need to adopt a formative approach to implementation and evaluation, reflecting, and learning along the way.

Taking account of the underlying principles, we have designed an implementation strategy for the GM CLAHRC that comprises four building blocks:

1. The PARIHS framework as an underpinning conceptual model recognizing the complexity and interplay of evidence, context, and facilitation;

2. A modified version of the Model for Improvement, providing an operational framework, with an actionable set of steps for implementation, but with inherent flexibility;

3. Multiprofessional implementation teams with designated roles of clinical leads, academic leads and knowledge transfer associates (KTAs) to lead, facilitate, and support the process of implementation;

4. Embedded evaluation and learning, in the form of cooperative inquiry and internal evaluation.

#### The PARIHS framework

**Figure 1:**
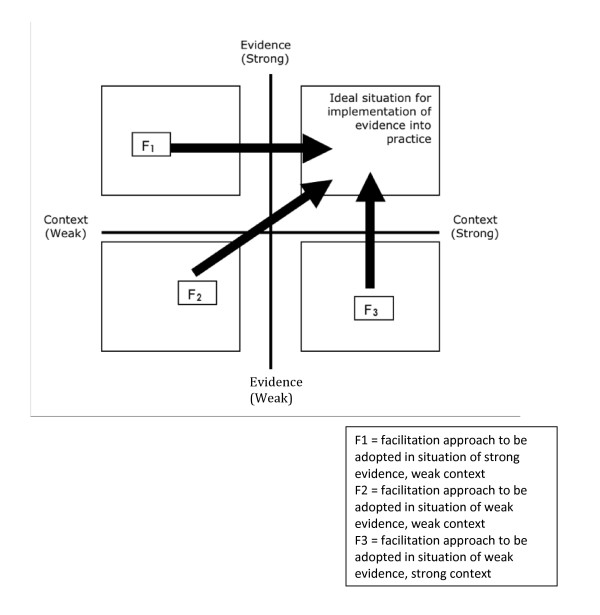
**The PARIHS Conceptual Framework **[28].

The PARIHS framework (Figure 1) proposes that the successful implementation of research evidence into practice is dependent on the complex interplay of the evidence to be implemented (how robust it is and how it fits with clinical, patient, and local experience), the local context in which implementation is to take place (the prevailing culture, leadership, and commitment to evaluation and learning), and the way in which the process is facilitated (how and by whom) [20]. Since its initial publication, the PARIHS framework has been used nationally and internationally as a heuristic to guide the application of research evidence into practice and as the conceptual underpinning of a variety of tools and frameworks to be used at the point of care delivery [45-48].

Each of the key concepts of evidence, context, and facilitation is recognised to be multi-factorial and can be represented along a continuum from low to high, with research uptake likely to be greatest when all of the three elements are located at the high end of the continuum. Concept analysis of the evidence construct proposed that evidence comprised four key sub-elements, namely, research, clinical experience, patient experience, and local information [18]. Where research evidence is 'high' (*i.e*., is rigorous/robust), but is not matched by a similarly high level of clinical consensus or does not meet with patients' needs and expectations, or perceived priorities at a local level, the process of translating research into practice will be more difficult. A similar concept analysis of the context construct [31] suggested that context comprised key elements of culture, leadership, and evaluation. In a situation where evidence is 'high' (as measured in terms of the strength of the research, clinical and patient experience, and local information), implementation will be more challenging where the culture is not conducive to change, the leadership is weak, and there is not a prevailing evaluative culture within the unit or organization.

Facilitation addresses the broader organizational dimensions of implementation and helps to create the optimal conditions for promoting the uptake of evidence into practice in the given context. Concept analysis of the facilitation dimension [42] has shown that individuals appointed as facilitators (*e.g*., project leads, educational outreach workers, or practice development facilitators) can take on a number of approaches to facilitation ranging from a largely task-focused, project manager role to a more holistic, enabling model where the facilitator works at the level of individuals, teams, and organizations to create and sustain a supportive context for evidence-based care (*e.g*., by analysing, reflecting, and changing attitudes, behaviours, and ways of working). The key to successful implementation is matching the role and skills of the facilitator to the specific needs of the situation. As illustrated in Figure 1, different approaches to facilitation are required, depending on the strength of the evidence to be implemented and the context in which implementation is to take place. So, for example, where the evidence is strong, but the context is weak or unsupportive (situation F1), the facilitator has to pay particular attention to contextual issues, such as identifying barriers to implementation, and introducing strategies to deal with these. These could include strategies to identify and support internal champions for change, securing explicit support and commitment from senior leadership, and creating effective processes for staff involvement, participation, and communication. Conversely, where the context is generally supportive of change, but the evidence is weak or disputed (situation F3), the facilitator needs to focus more on building consensus around the evidence to be implemented, *e.g*., by bringing together different stakeholder groups (such as clinicians, patients, managers, and commissioners) to review existing research evidence, share their own experiences, and reach agreement on the changes to be made. Consequently, skilled facilitators need to be able to move across different points of the facilitation continuum to meet the different requirements of individuals, teams, and organizations at different points in time. However, this requires facilitators to possess a sophisticated range of knowledge, including diagnostic skills (to assess the organizational context and the needs of individuals and teams), project management skills (planning and evaluating implementation activities), and interpersonal skills (building relationships, supporting individual, team and organizational development and learning, overcoming resistance to change).

The PARIHS approach represents a useful overarching conceptual framework that shows what aspects of the implementation process should be assessed and, if necessary, influenced by the teams to make their interventions successful. However, it provides little guidance about how the implementation process might unfold in practice, in what way the facilitation component of the framework could be institutionalised in the CLAHRC organizational structures and processes, and what should be done to create an organizational environment open to reflection, learning, and co-production of knowledge. This is why the GM CLAHRC implementation programme supplements the PARIHS framework with other concepts and methods described below.

#### The Model for Improvement

The Model for Improvement was developed by Langley *et al*., working within the Institute for Healthcare Improvement in the US [49], and is based around the plan-do-study-act (PDSA) cycle, which was initially utilised in industry. The PDSA cycle is linked in to the Model with three key questions, namely: What are we trying to accomplish? How will we know that a change is an improvement? What changes can we make that will result in the improvements that we seek? The use of this model over time to implement change is often referred to as rapid-cycle improvement, where a number of small PDSA cycles take place one after the other to generate continuous, incremental improvements in care. The Model for Improvement features in many current day approaches to healthcare improvement [50], including large scale, collaborative projects [51].

**Figure 2:**
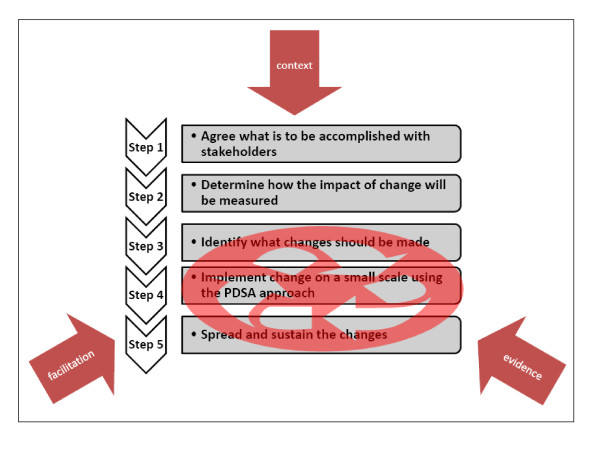
**GM CLAHRC approach to implementation: operational model embedded within the PARIHS framework**.

The basic elements of the Model for Improvement are represented in the operational framework that we use to guide implementation (Figure 2). This framework embeds the operational steps of the Model for Improvement within the conceptual coordinates of the PARIHS framework, emphasizing the need to consider the multi-dimensional elements of evidence and context and apply facilitation knowledge and skills to plan, undertake, and evaluate specific implementation interventions and projects. The Model for Improvement provides a useful supplement to the PARIHS framework by suggesting an iterative and reflective approach to implementation, emphasising the importance of non-linear cycles of activity and using an actionable set of incremental changes for putting previously discussed core principles of implementation into practice.

This modified version of the Model for Improvement shares some similarities with the existing frameworks, such as the knowledge-to-action cycle [27], but with less detail about how the different steps should be approached, enabling this to be determined by facilitators at a local level, depending upon their assessment of the local context. We elected to use this approach, rather than the existing knowledge transfer frameworks, because of the inherent flexibility that it allows, its focus on incremental improvement, and a special emphasis on planning for spread and sustainability of change. Due to its universality and flexibility, this approach can be applied to individual implementation projects run by the GM CLAHRC, but it does not specify what structures, roles, and processes should be deployed at an organizational level. These issues are addressed by the remaining two building blocks of the GM CLAHRC implementation strategy.

#### Multi-professional teams with designated roles to lead, facilitate, and support the implementation process

Central to the application of the PARIHS framework and the Model for Improvement are individuals in specific roles to facilitate implementation. Given the size, scope, and complexity of the GM CLAHRC implementation programme, we have adopted a team approach to fulfilling the required range of skills and knowledge needed, with the multiprofessional implementation teams comprising clinical leads, KTAs, academic leads, programme managers, and information analysts. Each of the four teams is led by an expert opinion leader [30], who has been appointed a clinical lead by virtue of their clinical expertise in the field (stroke, heart disease, diabetes, and chronic kidney disease) and their ability to influence colleagues about the evidence for change.

The knowledge transfer associates are appointments made specifically for CLAHRC, drawing on experience of knowledge transfer partnerships (KTPs) in both the private and public sectors in the UK [52]. Using a modified version of the KTP model we appointed two full-time KTAs per implementation theme (eight in total) who are engaged in a two-way transfer of knowledge between the implementation team and NHS organizations involved in the CLAHRC activities, and act as the main facilitators of change in the field. KTAs are supported by an academic lead, who provides the link to the implementation knowledge base, in keeping with the KTP model. In addition, each implementation team is supported by a programme manager, who works in a coordinating, overall project management role, and an information analyst, who supports work relating to data collection and analysis specific to individual programmes of work.

This team based approach to supporting the implementation process is important given the range of skills, knowledge, and experience that are necessary to implement, sustain, and spread the type of large-scale change the CLAHRC is aiming to achieve. In the language of communities of practice [35], we see this team-based approach as providing us with the boundary-spanning roles, objects, and practices that are needed to enable effective communication between the multiple communities involved in the CLAHRC and facilitate knowledge sharing and learning across boundaries. It offers an opportunity for different professional and organizational groups to participate in planning, undertaking, and evaluating the implementation projects, and thus engage in sharing, co-producing, and application of knowledge related to implementation.

#### Embedded evaluation and learning

The fourth building block relates to the strategy that we are adopting to evaluate the processes and outcomes of implementation as the work of the CLAHRC progresses. This is closely linked to the core principles upon which the overall implementation strategy is based. Ongoing learning, development, and reflection is built into the KTA role and the overall functioning of the implementation teams to ensure learning about implementation is systemically shared, collected, and analysed to add to the wider knowledge base about effective implementation. As highlighted above, each pair of KTAs is linked to a clinical and academic lead and supported by a programme manager. As multiprofessional implementation teams, these groups meet regularly to plan, deliver, and evaluate implementation strategies in practice. The KTAs also meet collectively for learning and sharing sessions, facilitated by one or more of the academic leads, to develop the sophisticated set of skills and knowledge required in the role, *e.g*., facilitation, project management, working with teams, and overcoming barriers to change. In addition, the KTAs meet on a monthly basis as a cooperative inquiry group, facilitated by an academic member of the CLAHRC team with expertise in action research.

A cooperative inquiry is a particular type of action research, whereby participants are treated as 'co-researchers' and participate in the 'thinking' and 'doing' of research [53], thus ensuring that the subject matter and subsequent findings are of direct relevance to those experiencing the problem. In the context of CLAHRC, the cooperative inquiry is seeking to explore how KTAs facilitate the implementation of research in the NHS to improve patient/client care and in the process develop their own understanding and practice in the facilitation of change. Cooperative inquiry was selected as an appropriate methodology because it is a way of researching with people who have related interests and experiences and who wish to examine with others how they might extend and deepen their understanding of their situation, bring about change, and learn how to improve their actions. Having a similar need to translate research findings into practice, the KTAs are researching together their experiences of facilitating the implementation of research and, in doing so, reconceptualise their understanding and also develop their skills in facilitation. In this way, the cooperative inquiry group provides a forum for the KTAs to develop their skills and seek support from peers, and a methodology for building new knowledge about the KTA role and approaches to implementation. The cooperative inquiry forms part of the internal evaluation strategy of the GM CLAHRC, consistent with our formative approach to implementation, whereby relevant evidence is tested and further enhanced as the implementation programme develops. Other internal evaluation activities include 'within' project evaluations, which in turn will feed into cross-case analyses of the various vascular-focused implementation projects [54]. We are also participating in the external evaluations of the CLAHRC initiative, funded by the National Institute for Health Research (NIHR) Service Delivery and Organization (SDO) programme.

#### Potential challenges to the GM CLAHRC approach to implementation

One challenge to the GM CLAHRC approach to implementation could be whether and how it is different to any other large-scale change management or quality improvement programme in healthcare, many of which have previously been criticised for insufficient attention to evidence [55] and lack of advanced theoretical thinking [13]. Within the approach we have outlined in this paper, our belief is that by embedding flexible, operational models for quality improvement within a conceptual framework of knowledge translation, we can draw on the strengths of both the evidence-based and quality improvement traditions [56].

Equally, questions could be asked as to why we have developed a programme of implementation activity, rather than a programme that is solely focused on implementation research. We support this course of action for a number of reasons. First, the CLAHRCs have an explicit remit to implement the findings from research in clinical practice and to increase the capacity of NHS organizations to engage with and apply research, as well as conducting high quality applied health research. Second, from initial discussions with the wide range of stakeholders involved in the GM CLAHRC, there was clearly an expressed need within local NHS organizations for support to implement existing research and address identified gaps between existing practice and recognised best practice. Third, we believe that by building knowledge about implementation as it is actually happening, with all the challenges and unpredictable issues that arise along the way, we can contribute to a deeper understanding about the realities of implementation in a large, complex health system. Within the discussion, we set out how we have drawn on existing evidence about implementation to support the use of a co-production model of research, embedding formative learning and evaluation strategies within the overall implementation approach of the GM CLAHRC, and thus ensuring that we add to the knowledge base about implementation as the work of the CLAHRC progresses.

Needless to say, there are many tensions inherent within the implementation approach we are taking, *e.g*., balancing local responsiveness and flexibility with maintaining an evidence-based approach to change and improvement, reconciling and coordinating the multiple roles, individuals, teams, and organizations involved in the implementation process, and integrating the formative learning from within the implementation programme with the wider CLAHRC strategy. These complexities that play out in the implementation programme in many ways mirror the complexities and challenges faced when attempting to translate research evidence into practice at a local level. Equally, it is important to recognise that CLAHRC implementation activity is not taking place in a vacuum. The NHS is undergoing a period of significant change, with major policy reforms, financial challenges, restructuring, reorganization, and the introduction of new commissioning arrangements [57]--all of which have knock-on effects on the work of the CLAHRC. These changes represent major contextual challenges at all levels of the healthcare system and have to be factored into the future planning and ongoing application of the GM CLAHRC implementation strategy. As our work progresses, we are recording and collating our practical experiences of applying the implementation models to individual projects within the broader cardiovascular theme. As the external environment is changing, we will, of necessity, have real-time opportunities to test out our principles of flexible, context-responsive implementation and evaluation approaches.

## Summary

The paper has described the types of evidence about implementation, set out the key principles of the GM CLAHRC implementation strategy, and discussed the conceptual and operational frameworks that have been selected, as well as the supporting resources and evaluation required to put this strategy into practice. In particular, it highlights the importance of an integrative conceptualisation of knowledge about implementation, the complexity of the implementation process, and the need for interventions that are situationally relevant, responsive, flexible, and collaborative. It also provides an example of a theory-informed approach to implementation, which combines a number of models, frameworks, and methods allowing the implementation of multi-faceted interventions targeted at different stakeholders, with a range of contextual factors and mechanisms of change.

## Competing interests

The authors declare that they have no competing interests.

## Authors' contributions

All authors contributed to the conception and design of the paper. GH prepared the initial draft of the manuscript and all authors were involved in the reviewing and revising process. All authors read and approved the final manuscript.
